# Tai Chi exercise improves sleep quality in older adults with mild insomnia by enhancing slow-wave activity during deep sleep: a 12-week randomized controlled trial

**DOI:** 10.3389/fphys.2026.1795646

**Published:** 2026-04-15

**Authors:** Mozhu Lyu, Jiaqi Cheng, Jiayu Li

**Affiliations:** 1 School of Martial Arts and Dance, Shenyang Sport University, Shenyang, Liaoning, China; 2 Department of Wushu and National Traditional Sports, Henan Sports University, Zhengzhou, Henan, China; 3 School of Martial Arts, Henan University, Zhengzhou, Henan, China

**Keywords:** older adults, sleep electroencephalography, sleep quality, slow-wave activity, Tai Chi

## Abstract

**Background:**

Sleep quality declines with age in older adults, and pharmacological interventions have multiple limitations. As a safe and accessible mind-body exercise, Tai Chi has preliminary evidence supporting its sleep-improving effects, but objective mechanistic studies based on sleep electroencephalography (EEG) remain scarce. Objective: This study aimed to clarify the effect of Tai Chi intervention on improving sleep quality in older adults and reveal its core mechanism through regulating sleep EEG characteristics, using a combination of questionnaires and sleep EEG monitoring.

**Methods:**

A randomized controlled trial design was adopted, enrolling 67 older adults aged 65–75 years with mild sleep disturbance, who were randomly divided into the Tai Chi group (n=33) and the control group (n=34). The Tai Chi group received 12 weeks of standardized 24-form Tai Chi training (5 times per week, 60 minutes per session), while the control group maintained daily activities without regular exercise. Subjective sleep quality was assessed using the Pittsburgh Sleep Quality Index (PSQI) and sleep diaries. Meanwhile, polysomnography (PSG) was used to collect sleep EEG data, analyzing sleep structure (proportion of each sleep stage) and EEG activity characteristics (slow-wave power, sleep spindle density).

**Results:**

After intervention, the total PSQI score and sleep onset latency in the Tai Chi group were significantly reduced, and sleep efficiency was significantly enhanced (*P* < 0.001). PSG results showed that the proportion of deep sleep stage (N3) in the Tai Chi group increased by 23.1%, delta wave power (0.5-4Hz) was significantly enhanced (*P* < 0.01), and sleep spindle density (N2 stage) increased by 6.0% (*P* = 0.07, marginally significant), while no significant changes were observed in sleep indicators of the control group. Correlation analysis revealed that the reduction in total PSQI score was significantly negatively correlated with the increase in N3 stage proportion (r=-0.464, *P* = 0.0065) and the enhancement of delta wave power (r=-0.382, *P* = 0.028) in the Tai Chi group.

**Conclusion:**

Twelve-week Tai Chi exercise intervention can significantly improve subjective and objective sleep quality in older adults. Its core mechanism is related to enhancing slow-wave activity during deep sleep and optimizing N2 stage sleep spindle density, providing objective EEG evidence for the non-pharmacological intervention of Tai Chi in sleep disorders of older adults.

## Introduction

The incidence of sleep disorders in older adults over 60 years old worldwide exceeds 50% (Tobias and Pisani, 2025), and chronic sleep disturbances are closely associated with cognitive decline and cardiovascular diseases, severely impairing their health-related quality of life (Tobias and Pisani, 2025; Schubert et al., 2002). Among existing non-pharmacological interventions, Tai Chi has become a research hotspot for intervening in sleep disorders of older adults due to its gentle movements and adaptability to the exercise capacity of older adults (Chan et al., 2022; Sun et al., 2025), but it lacks objective mechanistic evidence at the electroencephalography (EEG) level. The core methods for sleep quality assessment mainly include questionnaires (subjective assessment) and sleep EEG monitoring (objective assessment). The former can quickly obtain the subjective sleep experience of subjects, while the latter can accurately capture changes in sleep structure and EEG activity. The combination of the two can improve the scientificity and comprehensiveness of research conclusions (Pierson-Bartel and Ujma, 2024; Bitkina et al., 2022). However, most existing studies rely on subjective questionnaires to evaluate the sleep-improving effects of Tai Chi (Lei et al., 2025; Chen et al., 2025b), and only a few objective studies focus on the impact of Tai Chi on sleep structure (Wang et al., 2024). There is insufficient mechanistic exploration on core sleep EEG indicators (slow waves, sleep spindles), making it difficult to clarify the neuroelectrophysiological basis of Tai Chi’s sleep-improving effects.

Preliminary clinical studies have confirmed that Tai Chi intervention can improve subjective sleep quality in older adults, with a randomized controlled trial (RCT) and a large cross-sectional study both demonstrating positive effects of Tai Chi on sleep-related parameters and aging-related sleep deterioration (Irwin et al., 2008; Cheng et al., 2023). Recent meta-analyses have further defined the effective intervention dose of Tai Chi for sleep improvement, confirming that Tai Chi practice of 150–300 minutes per week for 8–16 weeks is the optimal dose for improving sleep quality in older adults (Lei et al., 2025; Chen et al., 2025b), and Tai Chi can thus be regarded as a priority intervention for community-dwelling older adults with sleep disturbances. However, currently only one study combining sleep questionnaires and polysomnography (PSG) explored the effect of 8-week Tai Chi exercise on sleep quality in older adults, showing that Tai Chi exercise may improve subjectively reported sleep quality, alleviate general drowsiness, extend sleep duration, and optimize the sleep process and structure (Wang et al., 2024).

Scholars generally agree that sleep EEG indicators are the objective core for evaluating sleep quality (Pierson-Bartel and Ujma, 2024; Bitkina et al., 2022), among which delta waves (0.5-4Hz) in the deep sleep stage (N3) and sleep spindles (11-16Hz) in the light sleep stage (N2) are key electrophysiological markers reflecting sleep quality in older adults (Schönauer and Pöhlchen, 2018; Carpentier et al., 2017). Delta wave power is closely related to sleep recovery and memory consolidation, and its age-related decline is the core electrophysiological mechanism of reduced deep sleep in older adults (Bitkina et al., 2022; Cajochen et al., 2006; Long et al., 2021; Crenshaw and Edinger, 1999), suggesting that delta wave power can be used as an objective indicator to assess the severity of sleep disorders in older adults. As a characteristic EEG activity in the N2 stage, sleep spindle density and amplitude directly reflect sleep stability (Schönauer and Pöhlchen, 2018), and can inhibit external interference and maintain sleep continuity. Studies have confirmed that abnormal sleep spindle density is an important electrophysiological reason for the easy awakening of older adults during sleep (Chatburn et al., 2021). Animal experimental studies have also provided mechanistic support for this association (Sawada et al., 2024). Regulation of EEG activity (e.g., delta waves, sleep spindles) can directly affect sleep quality, providing a theoretical basis for exploring the effect of Tai Chi on sleep EEG (Sawada et al., 2024).

Despite the preliminary confirmation of Tai Chi’s value in improving subjective sleep quality in older adults, existing research has obvious deficiencies: the disconnection between subjective benefit evaluation and objective mechanistic exploration, the lack of in-depth research on the association between Tai Chi and core sleep EEG indicators, and the predominance of cross-sectional designs that cannot clarify causal relationships. These gaps highlight the need for a randomized controlled trial to explore the regulatory effects of Tai Chi on sleep EEG indicators. Therefore, this study adopted a 12-week Tai Chi intervention design to investigate its effects on N3 stage delta wave power and N2 stage sleep spindle density in older adults with mild insomnia, and combined subjective sleep questionnaires to analyze the correlation between EEG indicator alterations and subjective sleep changes.

The study hypothesized that H1: Twelve-week Tai Chi intervention may lead to a reduction in PSQI scores in older adults and may improve subjective sleep quality; H2: Tai Chi intervention may increase the proportion of deep sleep (N3 stage), enhance delta wave power and sleep spindle density, and optimize sleep structure and EEG activity; H3: The improvement of subjective sleep quality may be correlated with the optimization of sleep EEG indicators.

## Methods and materials

### Participants

In the study participants were mainly recruited from three communities in Shenyang City, Liaoning Province, China. All three communities are urban residential communities for retired residents, with a high proportion of older adults aged 65–75 years, and the residents have similar living habits and socioeconomic status. Inclusion criteria: Older adults aged 65–75 years; PSQI score between 5 and 10 points (mild sleep disturbance) (Buysse et al., 1989; Morin et al., 2011); No regular exercise in the past 3 months (exercise <1 time per week, <30 minutes per session) and no history of Tai Chi practice; Clear consciousness and ability to cooperate with questionnaires and sleep EEG monitoring; No severe cardiovascular and cerebrovascular diseases, neurological diseases, or mental disorders. Exclusion criteria: Acute sleep disorders (course <3 months); Drug-dependent insomnia (long-term use of sedative-hypnotic drugs); Contraindications to PSG; Cognitive impairment (MoCA score <20 points); Persistent daytime napping behavior (≥1 nap per day and duration ≥30 min); Extreme chronotype; Inability to adhere to the 12-week intervention.

Taking the change in total PSQI score as the primary outcome indicator, referring to previous studies (Lei et al., 2025) (α=0.05, β=0.2, effect size≈0.6), each group required 33 cases. Considering a 15% attrition rate, a total of 80 subjects were recruited in this trial, including 7 dropouts in the Tai Chi group and 6 dropouts in the control group. Finally, 33 subjects were in the Tai Chi group and 34 in the control group. This study was approved by the Ethics Committee of Shenyang Sport University, and all subjects signed written informed consent forms, in line with the ethical requirements of the Declaration of Helsinki.

There were no significant differences in baseline data (gender, age, educational level, Body Mass Index (BMI), total PSQI and Montreal Cognitive Assessment (MoCA) scores, etc.) between the two groups (*P >*0.05), indicating comparability (**Table 1**).

**Table 1:** Comparison of baseline data between the two groups (x ± s or n, %).

Indicators	Tai Chi group (n=33)	Control group (n=34)	χ²/t	*P* values
Gender (male/female, n)	15/18	16/18	0.011	0.916
Age (years)	70.4 ± 2.7	69.9 ± 2.9	0.660	0.511
Educational level (n, %)			0.227	0.892
Primary school and below	8 (24.2%)	10 (29.4%)		
Junior high school	14 (42.4%)	13 (38.2%)		
Senior high school and above	11 (33.3%)	11 (32.4%)		
Height (cm)	167.6 ± 6.2	168.3 ± 6.5	0.405	0.686
Weight (kg)	62.6 ± 6.5	63.5 ± 6.9	1.059	0.293
BMI (kg/m²)	22.3 ± 1.1	22.5 ± 1.3	0.507	0.613
Total PSQI score	7.9 ± 2.0	8.2 ± 1.4	0.620	0.784
Total MoCA score	26.6 ± 2.0	27.0 ± 1.6	0.813	0.419
Physical activity level (MET-min/week)	582.2 ± 92.8	575.1 ± 77.4	0.346	0.730
Medication history (sleep-related drugs, n)	0/33	0/34	NA	NA
Underlying diseases (n, %)			0.219	0.639
Mild hypertension	(6, 18.2%)	(7, 20.6%)		
Type 2 diabetes	(4, 12.1%)	(3, 8.8%)		

NA represents not applicable.

### Study design

This study adopted a RCT design, with assessors (questionnaire scorers and EEG data analysts) being single-blind. A computer-generated random number table method was used for randomization. After screening and enrolling eligible participants, an independent researcher who was not involved in the subsequent intervention and assessment assigned the participants to the Tai Chi group or the control group at a 1:1 ratio according to the random number table, and the allocation sequence was concealed in sealed opaque envelopes until the start of the intervention. None of the residents in the three communities had participated in formal Tai Chi training or regular Tai Chi practice before the study. For the Tai Chi group, 24-form Tai Chi was adopted, taught standardized by senior Tai Chi coaches with national first-class social sports instructor qualifications. The practice was conducted 5 times a week (Monday to Friday), 60 minutes per session (including 10 minutes of warm-up, 40 minutes of movement practice, and 10 minutes of relaxation), with an intervention period of 12 weeks. Practice compliance was recorded throughout the study (required compliance ≥80%). For the control group, maintained daily living habits without participating in any regular exercise or sleep intervention. Follow-up was conducted every 4 weeks during the intervention period to remind them to maintain their original living conditions and avoid contact with Tai Chi-related practice.

### Assessment time points, intervention monitoring, and quality control

1. Assessment time points: Questionnaires and sleep EEG monitoring were conducted before intervention (Pre) and at the end of intervention (Post). Meanwhile, practice compliance of the Tai Chi group and adverse events of both groups were recorded. Mid-term follow-up was conducted every 4 weeks during the intervention period to check the practice compliance of the Tai Chi group and timely address insufficient compliance.
2. Tai Chi practice monitoring: To ensure the adherence of participants to the Tai Chi practice regimen (5 times/week for 12 weeks), we adopted a multi-dimensional adherence management strategy with the participation of community leaders: a) Community leaders assisted in publicity and mobilization before the intervention, and supervised the participants’ attendance during the intervention; b) Researchers conducted on-site attendance recording for each practice session, and the required compliance rate was ≥80%; c) Mid-term follow-up was conducted every 4 weeks, and for participants with low attendance, researchers and community leaders conducted one-on-one communication to understand the reasons and provide personalized solutions; d) A WeChat group was established for the Tai Chi group, where coaches released practice videos and reminders, and participants shared their practice experience to enhance motivation. The final average compliance rate of the Tai Chi group was 92.7%, which was significantly higher than the required 80%.
3. Comprehensive quality control was implemented throughout the study, including the following key aspects: Tai Chi coaches must have national first-class social sports instructor qualifications and pass standardized and specialized training and assessment on 24-form Tai Chi movements before taking office; Questionnaire assessors and EEG data analysts received special training. Instrument and data quality control: PSGs were calibrated before each use; EEG data were independently analyzed by 2 researchers; Questionnaire data were double-entered and verified by two persons to eliminate errors.

### Assessment indicators and methods

#### Subjective sleep quality assessment

PSQI: Including 7 dimensions and 18 items, such as sleep quality. The total score ranges from 0 to 21 points, with higher scores indicating poorer sleep quality. A score of 5–10 points suggests mild sleep disturbance. Sleep diary: Recorded continuously for 7 days, mainly recording the number of nocturnal awakenings (NNA), used to supplement the assessment of subjective sleep changes.

#### Objective sleep EEG monitoring

A portable PSG (SOMNOscreen™ plus, SOMNO medics, Germany) was administered in participants’ homes to ensure a natural sleep environment, recording EEG activity for ≥7 hours. EEG electrodes were placed at FP1, FPz, and FP2 sites according to the international 10–20 EEG system, with conductive electrode paste applied to optimize signal conduction and reduce impedance (target impedance <5 kΩ). According to the American Academy of Sleep Medicine (AASM) criteria (Silber, 2012), sleep structure was divided into wakefulness (W), light sleep (N1, N2), deep sleep (N3), and rapid eye movement (REM) sleep by a DOMINO Software. This study focused on the N3 stage, calculating the proportion of N3 stage in total sleep time, sleep onset latency (SOL), and sleep efficiency (SE). Core sleep EEG indicators were extracted, including N3 stage delta wave (0.5-4Hz) power and N2 stage sleep spindle (11-16Hz) density, which were processed and analyzed offline using the DOMINO Software.

### General and safety indicators

Baseline data such as age, gender, educational level, underlying diseases, and medication history of subjects were collected. The underlying diseases of the participants were mainly mild hypertension and type 2 diabetes with well-controlled blood pressure/blood glucose, and no participants had severe underlying diseases that affected sleep and exercise. The distribution of underlying diseases was not significantly different between the two groups (**Table 1**). Adverse events (such as muscle soreness, dizziness, etc.) during the intervention were recorded to assess the tolerability and safety of Tai Chi intervention.

### Statistical analysis

SPSS 26.0 software was used for data analysis. The Shapiro-Wilk test was used to test data normality. The data in this study were normally distributed. Measurement data were expressed as mean ± standard deviation (x ± s). Paired t-tests were used for intra-group comparisons before and after intervention, and independent samples t-tests were used for intergroup comparisons. Count data were expressed as rates (%), and χ² tests were used for intergroup comparisons. Repeated-measures analysis of variance (repeated-measures ANOVA) was used to analyze the group×time interaction effect and main effects, with *post-hoc* tests using Sidak correction. Partial eta squared (η_p_^2^) is reported as the effect size for ANOVA, with values of 0.01, 0.06, and 0.14 generally considered small, medium, and large effects, respectively. Spearman correlation analysis was used to explore the correlation between changes of subjective sleep scores and EEG indicators. *P* < 0.05 was considered statistically significant. Effect sizes were calculated using Cohen’s d, with d < 0.2 defined as tiny effect, 0.2 ≤ d < 0.5 as small effect, 0.5 ≤ d < 0.8 as medium effect, and d ≥ 0.8 as large effect.

## Results

### Safety indicators

During the 12-week intervention, a small number of mild adverse events occurred in the Tai Chi group, including 5 cases of muscle soreness (15.2%) and 2 cases of mild dizziness (6.1%), all of which were relieved after rest and adjusting the practice intensity and no severe adverse events occurred. No adverse events related to the intervention were found in the control group. The overall adverse event rate of the Tai Chi group was 21.3%, and all participants tolerated the Tai Chi intervention well.

### Changes in subjective sleep quality before and after intervention in both groups

**Figure 1:**
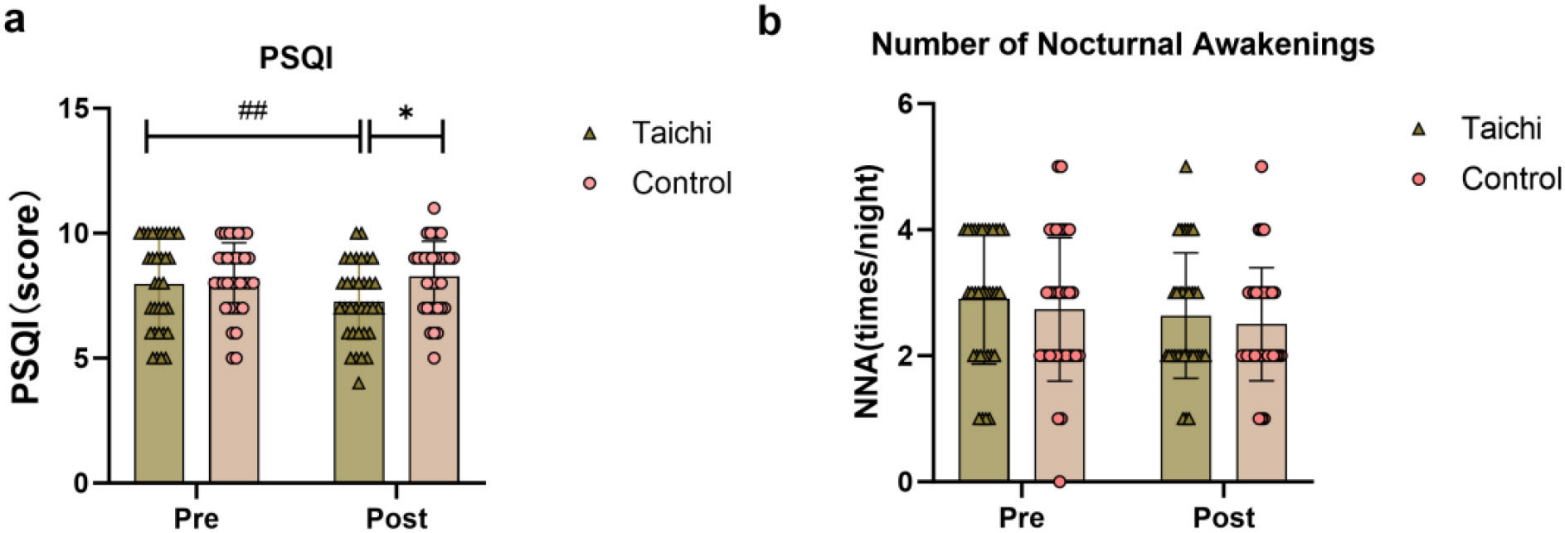
Comparison of changes in subjective sleep quality indicators between the two groups before and after intervention. ## indicates comparison of indicators in the Tai Chi group before and after intervention (*P* < 0.01); * indicates a significant difference between the Tai Chi group and the Control group at post-intervention (*P* < 0.05). Panel **(a)** shows PSQI scores with Taichi group decreasing post-intervention and significant differences indicated. Panel **(b)** shows number of nocturnal awakenings, with both groups having similar means before and after intervention. Triangles represent Taichi, circles represent control, and error bars show variability.

The changes in subjective sleep quality indicators of the two groups are shown in **Figure 1**. Repeated-measures ANOVA showed that there was a significant group×time interaction effect (F(1,65)=8.307, *P* = 0.0053,η_p_^2^ = 0.113) and time main effect (F(1,65)=5.922, *P* = 0.0177, η_p_^2^ = 0.084) for total PSQI score, but no significant group main effect (*P >*0.05). There was no significant difference in PSQI scores between the two groups before intervention (*P >*0.05). After intervention, the total PSQI score of the Tai Chi group was significantly lower than that before intervention. Additionally, the PSQI score of the Tai Chi group was significantly lower than that of the control group after intervention (*P* < 0.05). There was no significant change in the number of nocturnal awakenings (**Table 2**).

**Table 2:** Comparison of changes in subjective sleep quality indicators between the two groups before and after intervention (x ± s).

Indicators	Groups	Pre	Post	t_1_	*P*_1_ values	Cohen’s d_1_
Total PSQI score	Tai Chi (n=33)	7.9 ± 2.0	7.2 ± 1.5^##^	3.731	0.0008	0.65
	Control (n=34)	8.2 ± 1.4	8.3 ± 1.4^*^	0.310	0.941	0.05
	t_2_	0.620	2.605			
	*P*_2_ values	0.784	0.0204			
	Cohen’s d_2_	0.15	0.64			
NNA (times/night)	Tai Chi (n=33)	2.9 ± 1.1	2.3 ± 0.9	2.281	0.051	0.40
	Control (n=34)	2.7 ± 1.1	2.4 ± 0.8	1.998	0.097	0.34
	t_2_	0.696	0.546			
	*P*_2_ values	0.737	0.828			
	Cohen’s d_2_	0.17	0.13			

t_1,_ *P*_1_, and d_1_ are the results of paired t-tests for intra-group comparisons before and after intervention; t_2,_ *P*_2_, and d_2_ are the results of independent samples t-tests for intergroup comparisons after intervention; ## indicates a significant difference within the Tai Chi group (pre vs. post intervention, *P* < 0.01); * indicates a significant difference between the Tai Chi group and the Control group at post-intervention (*P* < 0.05).

### Changes in sleep EEG indicators before and after intervention in both groups sleep structure indicators

**Figure 2:**
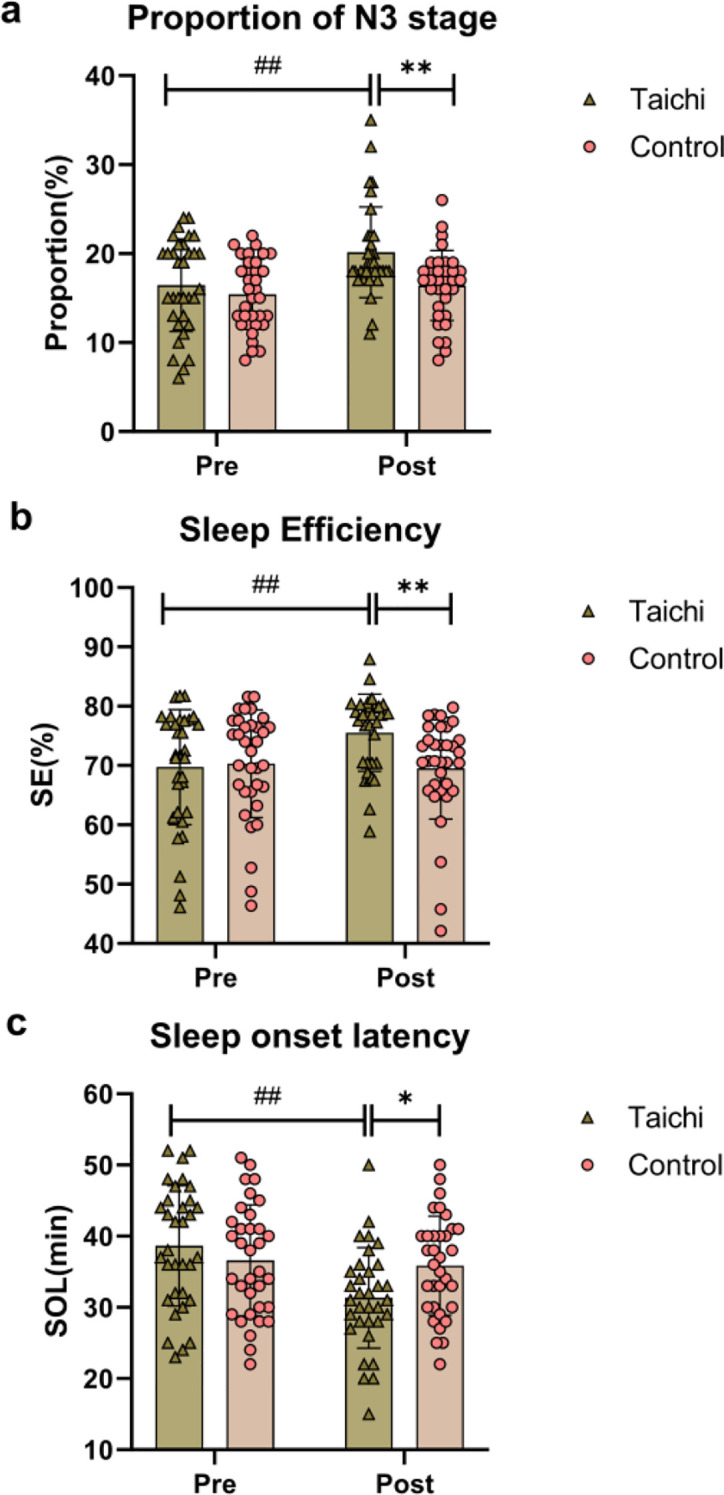
Comparison of changes in Proportion of N3 stage **(a)**, sleep efficiency **(b)**, and sleep latency **(c)** between the two groups before and after intervention. ## indicates comparison of the Tai Chi group before and after intervention, *P* < 0.01; * and ** indicate comparisons between the Tai Chi group and the Control group after intervention, *P* < 0.05 and *P* < 0.01, respectively.

The changes in the proportion of N3 stage, SE and SOL of the two groups are shown in **Figure 2**. Repeated-measures ANOVA showed that there was a significant group×time interaction effect (F(1,65)=5.361, *P* = 0.0238, η_p_^2^ = 0.076) and time main effect (F(1,65)=16.26, *P* = 0.0001, η_p_^2^ = 0.20) for the proportion of N3 stage. After intervention, the proportion of N3 stage in the Tai Chi group was significantly increased by 23.1% compared with that before intervention(*P* < 0.0001), and was significantly higher than that in the control group(*P* = 0.002), while there was no significant change in the control group (*P >*0.05).

SE (F (1, 65) = 12.99, *P* = 0.0006, η_p_^2^ = 0.167) and SOL (F (1, 65) = 16.96, *P* = 0.0001, η_p_^2^ = 0.207) also had significant group×time interaction effects. After intervention, the sleep efficiency of the Tai Chi group was significantly improved compared with that before intervention (*P* < 0.0001), and the SOL was significantly shortened compared with that before intervention (*P* < 0.0001), both of which were significantly better than those in the control group (*P* < 0.05), while there was no significant change in the control group (*P >*0.05) (**Table 3**).

**Table 3:** Comparison of changes in sleep structure indicators between the two groups before and after intervention (x ± s).

Indicators	Groups	Pre	Post	t_1_	*P*_1_ values	Cohen’s d_1_
Proportion of N3 stage (%)	Tai Chi (n=33)	16.4 ± 5.2	20.2 ± 5.1^##^	4.445	<0.0001	0.77
	Control (n=34)	15.4 ± 3.9	16.4 ± 3.7^**^	1.223	0.400	0.21
	t_2_	0.932	3.344			
	*P*_2_ values	0.581	0.002			
	Cohen’s d_2_	0.23	0.82			
SE (%)	Tai Chi (n=33)	69.7 ± 9.7	75.5 ± 6.5^##^	4.473	<0.0001	0.78
	Control (n=34)	70.3 ± 9.1	69.6 ± 8.7^**^	0.595	0.800	0.10
	t_2_	0.271	2.860			
	*P*_2_ values	0.954	0.0098			
	Cohen’s d_2_	0.07	0.70			
SOL (minutes)	Tai Chi (n=33)	38.6 ± 8.5	31.3 ± 7.1^##^	6.458	<0.0001	1.12
	Control (n=34)	36.6 ± 7.8	35.9 ± 6.9^*^	0.686	0.745	0.12
	t_2_	1.088	2.436			
	*P*_2_ values	0.479	0.032			
	Cohen’s d_2_	0.27	0.59			

Sleep structure indicators are based on PSG data, determined in accordance with AASM criteria; SE = total sleep time/time in bed × 100%; t_1,_ *P*_1_, and d_1_: results of paired t-tests for intra-group comparisons before and after intervention; t_2,_ *P*_2_, and d_2_: results of independent samples t-tests for intergroup comparisons after intervention; ## indicates a significant difference in the Tai Chi group before and after intervention (*P* < 0.01); * and ** indicate significant differences between the Tai Chi group and the Control group after intervention (*P* < 0.05 and *P* < 0.01, respectively).

### EEG activity indicators

**Figure 3:**
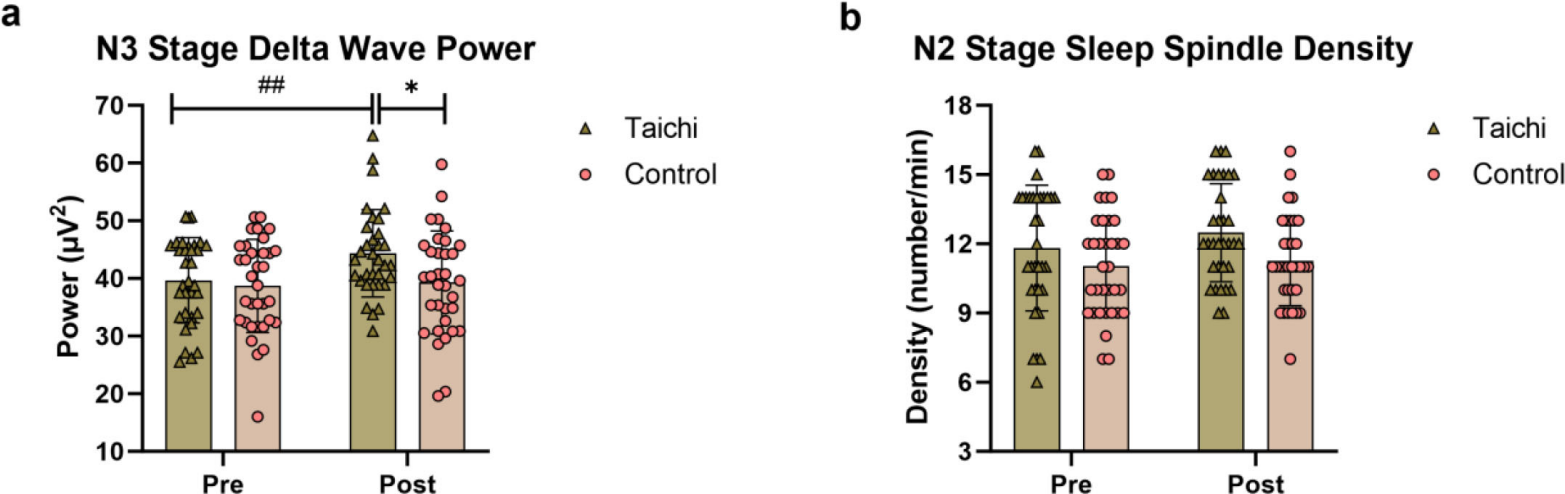
Comparison of changes in N3 stage delta wave power **(a)** and N2 stage sleep spindle density **(b)** between the two groups before and after intervention ## indicates comparison of the Tai Chi group before and after intervention, *P* < 0.01; * indicates comparison between the Tai Chi group and the Control group after intervention, *P* < 0.05.

The changes in N3 stage delta wave power and N2 stage sleep spindle density of the two groups are shown in **Figure 3**. Repeated-measures ANOVA showed that there was a significant group×time interaction effect (F(1,65)=4.156, P = 0.0456, η_p_^2^ = 0.06) and time main effect (F(1,65)=6.868, *P* = 0.0109, η_p_^2^ = 0.096) for N3 stage delta wave power. After 12 weeks of Tai Chi intervention, the delta wave power of the subjects was significantly increased compared with that before intervention (*P* = 0.0034), and was significantly higher than that of the control group (*P* = 0.023), while there was no significant change in the control group (*P >*0.05).

Regarding N2 stage sleep spindle density, although the Tai Chi group showed no significant increase, it had a marginal effect (*P* = 0.07), and there was no significant change in the control group (*P*>0.05). There was no statistically significant difference between the two groups after intervention (*P*>0.05) (**Table 4**).

**Table 4:** Comparison of changes in sleep EEG activity indicators between the two groups before and after intervention (x ± s).

Indicators	Groups	Pre	Post	t_1_	*P*_1_ values	Cohen’s d_1_
N3 stage delta wave power (μV²)	Tai Chi (n=33)	39.7 ± 7.4	44.3 ± 7.6^##^	3.270	0.0034	0.57
	Control (n=34)	38.7 ± 8.1	39.3 ± 9.3^*^	0.414	0.897	0.07
	t_2_	0.483	2.562			
	*P*_2_ values	0.862	0.023			
	Cohen’s d	0.12	0.63			
N2 stage sleep spindle density (number/minute)	Tai Chi (n=33)	11.8 ± 2.7	12.5 ± 2.1	2.144	0.07	0.37
	Control (n=34)	11.0 ± 2.2	11.3 ± 1.9	0.768	0.692	0.13
	t_2_	1.430	2.211			
	*P*_2_ values	0.286	0.056			
	Cohen’s d_2_	0.35	0.54			

Delta wave frequency range: 0.5–4 Hz; Sleep spindle frequency range: 11–16 Hz; t_1,_ *P*_1_, and d_1_: results of paired t-tests for intra-group comparisons before and after intervention; t_2,_ *P*_2_, and d_2_: results of independent samples t-tests for intergroup comparisons after intervention; ## indicates a significant difference in the Tai Chi group before and after intervention (*P* < 0.01); * indicates a significant difference between the Tai Chi group and the Control group after intervention (*P* < 0.05).

### Correlation between subjective sleep quality and EEG indicators

**Figure 4:**
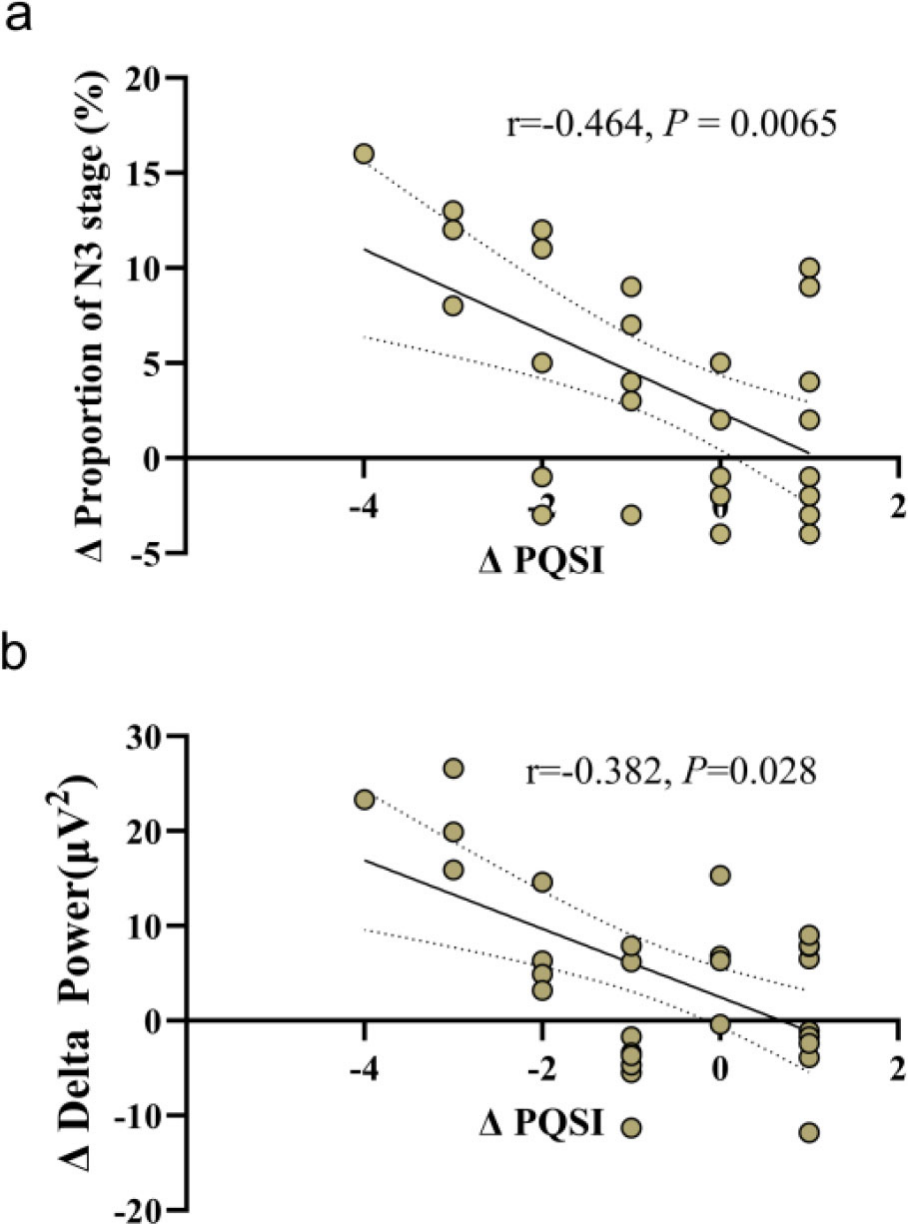
Negative correlations between the change in total PSQI score and the change in proportion of N3 stage **(a)** and the change in delta wave power during N3 stage **(b)** after intervention in the Tai Chi group.

Spearman correlation analysis showed that the reduction in total PSQI score after intervention in the Tai Chi group was significantly negatively correlated with the increase in N3 stage proportion and the enhancement of delta wave power (**Figure 4**), suggesting that the improvement of subjective sleep quality is closely related to the optimization of objective EEG indicators.

## Discussion

This randomized controlled trial confirmed that a 12-week standardized 24-form Tai Chi intervention, 5 times a week for 60 minutes each time, can significantly improve the subjective and objective sleep quality of older adults aged 65–75 with mild sleep disturbance.

### The effect of Tai Chi on improving subjective sleep quality in older adults

This study confirmed through PSQI and sleep diaries that 12-week Tai Chi intervention can significantly improve the subjective sleep quality of older adults, characterized by improved sleep quality, and the number of nocturnal awakenings showed a decreasing trend without statistical significance (*P* = 0.051), which is consistent with the results of several studies (Chen et al., 2025b; Siu et al., 2021; Li et al., 2020). We speculate that this is related to the high-intensity intervention dose of “5 times a week, 60 minutes per session” adopted in this study. In addition, this study realized the dynamic tracking of subjective sleep changes through daily sleep diaries, making up for the deficiency of only two static assessments before and after intervention, and more accurately captured the progressive improvement of indicators such as sleep quality and the number of nocturnal awakenings. Furthermore, unlike studies without controls, this study used a control group that did not participate in regular exercise as a reference, excluding the interference of exercise placebo effect on subjective scores, confirming that the subjective sleep improvement effect of Tai Chi is not only derived from the behavioral intervention of regular exercise but also related to the unique mind-body synergy of Tai Chi. The gentle movements and abdominal breathing of Tai Chi can activate the parasympathetic nervous system (Gao et al., 2024), alleviate daytime anxiety and physical tension (Chen et al., 2025a; Kuang et al., 2023), thereby improving the subjective sleep experience. Meanwhile, the behavioral habits formed by regular practice can stabilize the sleep rhythm and improve sleep satisfaction. This result not only echoes existing studies on the subjective efficacy of Tai Chi but also is consistent with the findings of previous studies on non-pharmacological interventions for sleep disorders (Voiß et al., 2019), suggesting that Tai Chi may be a potential effective non-pharmacological intervention for improving subjective sleep quality in older adults. In addition to the direct effect of Tai Chi’s mind-body synergy on sleep, the social and daily life context of the participants also contributed to the improvement of subjective sleep quality. The group Tai Chi practice in the community provided a social interaction platform for the older adults, reducing their loneliness and social isolation in daily life (Chan et al., 2022). Meanwhile, the regular practice schedule formed a stable daily routine for the participants, regulating their circadian rhythm and reducing the irregularity of sleep-wake time caused by idle retirement life (Fan et al., 2025). The combination of social interaction and regular daily life, together with the physical and psychological regulation of Tai Chi, formed a synergistic effect, which further improved the participants’ subjective sleep experience and sleep satisfaction.

### The EEG mechanism of Tai Chi in improving sleep

#### Enhancing slow-wave activity during deep sleep

This study found that Tai Chi intervention can significantly increase the proportion of N3 stage and N3 stage delta wave power, and the enhancement of delta wave power is significantly negatively correlated with the improvement of total PSQI score, filling the gap in the association between delta wave regulation and subjective sleep improvement. Delta waves are the core EEG activity during deep sleep, closely related to sleep recovery and memory consolidation (Cajochen et al., 2006; Long et al., 2021). The decline of delta wave power in older adults is an important electrophysiological feature of sleep quality deterioration (Long et al., 2021; Crenshaw and Edinger, 1999), and the regulatory effect of Tai Chi on delta waves may be the key to its improvement of sleep quality in older adults.

Compared with conventional aerobic exercises (such as walking), Tai Chi may have more advantages in regulating deep sleep and delta waves. This may be related to the mind-body synergy of movement rhythm and breathing regulation of Tai Chi. Conventional exercises focus more on physical activity, while the gentle movements and abdominal breathing of Tai Chi can synchronously inhibit sympathetic nerve excitation (Wei et al., 2016; Teng et al., 2025), promote the coordinated activity of the cortico-thalamic network (Cui et al., 2021), and thus more efficiently enhance delta wave generation (Si et al., 2019). In addition, compared with mindfulness meditation (Cahn and Polich, 2006), Tai Chi achieves better deep sleep improvement than single psychological intervention through dual physical and psychological regulation, further highlighting its clinical advantages.

#### Optimizing N2 stage sleep spindle density

Sleep spindles (N2 stage, 11-16Hz) can inhibit external interference and maintain sleep stability. The decrease in sleep spindle density is the core electrophysiological reason for the easy awakening and decreased continuity of older adults during sleep (Bragazzi et al., 2025). This study found that 12-week Tai Chi intervention induced a non-significant increasing trend in sleep spindle density by 6.0% (*P* = 0.07, Cohen’s d=0.37, small effect). The small effect size and the relatively small sample size may jointly contribute to the lack of statistical significance, and further large-sample studies are needed to verify this trend. Studies have pointed out that sleep spindle generation depends on the coordinated activity of the thalamocortical circuit (Carpentier et al., 2017; Fang et al., 2017). The decline of thalamic neuron function in older adults is prone to lead to a decrease in sleep spindle density, thereby causing sleep fragmentation. This mechanism provides a theoretical basis for exploring the regulatory effect of intervention methods on sleep spindles (Fang et al., 2017). Currently, there is a lack of research on the association between Tai Chi and sleep spindle regulation. There are no studies on the effect of Tai Chi practice on sleep spindle density during sleep, nor in-depth exploration of its regulatory mechanism. Existing limited evidence suggests that Tai Chi may have potential regulatory effects on sleep spindles, and its effect on sleep spindles needs to be further compared with other non-pharmacological interventions in future head-to-head studies. The underlying mechanism may be attributed to the synergistic interplay between the rhythmic movements, regulated breathing, and mindfulness-oriented focus inherent in Tai Chi practice: specifically, this multimodal intervention activates the parasympathetic nervous system, suppresses excessive excitability of the thalamocortical circuit, and consequently enhances sleep spindle generation (Fang et al., 2017)—a key electrophysiological correlate of sleep stability—thereby reducing nocturnal awakenings and improving overall sleep continuity.

### Correlation between subjective and objective indicators

This study confirmed through correlation analysis that the improvement of subjective sleep quality is significantly correlated with the increase in proportion of N3 stage sleep and N3 stage delta wave power, confirming that Tai Chi can synchronously improve the subjective sleep experience and objective sleep quality of older adults by regulating sleep EEG activity, forming a synergistic improvement effect, and filling the gap in the connection between subjective benefits and objective mechanisms in the field of Tai Chi. This result is consistent with the review conclusion by Fang et al. (2017), that is, the optimization of core EEG indicators is an important physiological basis for the improvement of subjective sleep quality. This study is the first to verify this association in the context of Tai Chi intervention. From the mechanistic perspective, the rhythmic movements of Tai Chi may synchronously regulate the excitability of the cerebral cortex, could promote the coordinated activity of the cortico-thalamic network, thereby may enhance delta wave generation, prolonging deep sleep duration, and improving sleep recovery efficiency (Cui et al., 2021; Si et al., 2019). This mechanism may be different from the mode of conventional exercise inducing deep sleep through physical fatigue and the path of mindfulness meditation improving sleep through psychological relaxation (Kim et al., 2024; Buela-Casal et al., 2025; Gong et al., 2016), providing a unique electrophysiological mechanism support for the non-pharmacological intervention of Tai Chi in sleep disorders of older adults, and further highlighting its clinical advantages in the intervention of sleep in older adults.

### Study limitations and further research

Despite obtaining some interesting research results, this study still has the following limitations: (1) The sample size is limited to a single region, and its representativeness may be insufficient; (2) No different Tai Chi practice dose groups were set up, so the optimal intervention dose cannot be clarified; (3) The follow-up period is short (12 weeks), and the long-term durability of the curative effect was not evaluated; (4) EEG analysis only focused on core indicators, without involving more refined brain network connection analysis.

Several avenues for future research are proposed to address the limitations of the present study and advance the field: firstly, conduct multi-center large-sample studies to enhance the representativeness of study participants; secondly, establish different Tai Chi practice dose groups (e.g., 3 times vs. 5 times per week) to investigate the dose-response relationship between Tai Chi practice and sleep improvement; additionally, extend the follow-up period to 6–12 months to assess the long-term durability of the intervention effect; and finally, integrate resting-state EEG analysis to further explore the impacts of Tai Chi on sleep regulation-related brain networks, thereby enriching the mechanistic insights into Tai Chi’s sleep-beneficial effects.

## Conclusion

Twelve-week standardized Tai Chi intervention can significantly improve the subjective and objective sleep quality of older adults. The core mechanisms are: increasing delta wave power and N3 stage proportion during deep sleep to enhance sleep recovery function; optimizing N2 stage sleep spindle density, which may have a potential effect on improving sleep continuity and needs to be further verified by large-sample studies. The improvement of subjective sleep quality in older adults is significantly correlated with the optimization of sleep EEG indicators, confirming that Tai Chi can comprehensively improve sleep quality by regulating sleep EEG activity, providing dual subjective and objective evidence for the non-pharmacological intervention of Tai Chi in sleep disorders of older adults, and having important clinical application value and promotion significance.

## Data Availability

The raw data supporting the conclusions of this article will be made available by the authors, without undue reservation.
